# Characterization of the TCR V repertoire of piperacillin-specific T-cells

**DOI:** 10.1186/2045-7022-4-S3-P39

**Published:** 2014-07-18

**Authors:** Zaid Al-Attar, Lee Faulkner, John Farrell, Paul Whitaker, Daniel Peckham, Kevin Park, Dean Naisbitt

**Affiliations:** 1 University of Liverpool, MRC Centre for Drug Safety Science, UK; 2 St James's Hospital, Regional Adult Cystic Fibrosis Unit, UK

## Background

Piperacillin is a -lactam antibiotic that is frequently used to combat bacterial infections in patients with cystic fibrosis. However, its use is associated with a high incidence of delayed-type hypersensitivity reactions. Our previous studies have shown lymphocyte proliferative responses and cytokine secretion from PBMCs in approximately 75% of hypersensitive patients. In contrast, PBMCs from tolerant controls were not activated by these antibiotics. Drug-responsive T-cell clones isolated from piperacillin hypersensitive patients were CD4+ and the drug derived antigen was presented by HLA class II molecules. These data highlight the pivotal role of T cells in mediating the piperacillin hypersensitivity, however, little is known about TCR V usage. Our project aims to explore the phenotype of T cells involved in piperacillin hypersensitivity looking at the TCR V usage, chemokine receptor expression and cytokine release in both hypersensitive patients and in drug naïve healthy donors.

## Methods

PBMCs from hypersensitive patients were cultured in the presence of piperacillin and drug-responsive T-cell clones were generated by serial dilution. TCR V usage and chemokine receptor expression were analyzed by flow cytometry. Cytokine release was assessed by ELISpot. Naïve T cells from healthy donors were primed to piperacillin using dendritic cells to present the drug antigen.

## Results

A total of 20 piperacillin-specific clones were generated from the hypersensitive patients. Most of the clones were CD4+ and drug stimulation was associated with the secretion of IFNγ (85% of the clones), IL5 (65%), IL13 (35%), perforin (60%), granzyme-B (25%) and FasL (65%). Clones expressed high levels of CCR8, CCR4, CCR10, CXCR3, CCR2 and CCR9. TCR V usage was by these clones was diverse. Piperacillin-specific T-cell proliferative responses and cytokine secretion were detected when naïve T-cells were primed with piperacillin for 7 days. TCR V usage was diverse with marked expansion of TCR V9 and V13.2 on memory T-cells post priming. CDR3 spectratyping analysis will be used to characterize whether expansion of the individual TCR Vβs is polyclonal or oligoclonal. In on-going experiments we are cloning T-cells from healthy volunteers to analyse TCR V expression.

## Conclusion

These data show that piperacillin-responsive CD4+ T-cells express a diverse repertoire of TCR Vβs.

